# A Panel of Recombinant Mucins Carrying a Repertoire of Sialylated *O*-Glycans Based on Different Core Chains for Studies of Glycan Binding Proteins

**DOI:** 10.3390/biom5031810

**Published:** 2015-08-12

**Authors:** Reeja Maria Cherian, Chunsheng Jin, Jining Liu, Niclas G. Karlsson, Jan Holgersson

**Affiliations:** 1 Department of Clinical Chemistry and Transfusion Medicine, Institute of Biomedicine, Sahlgrenska Academy, University of Gothenburg, SE-41345, Gothenburg 41390, Sweden; E-Mails: jining.liu@gu.se (J.L.); jan.holgersson@clinchem.gu.se (J.H.); 2 Department of Medical Biochemistry, Institute of Biomedicine, Sahlgrenska Academy, University of Gothenburg, SE-40530, Gothenburg 41390, Sweden; E-Mails: chunsheng.jin@medkem.gu.se (C.J.); niclas.karlsson@medkem.gu.se (N.G.K.)

**Keywords:** *O*-glycans, sialic acid, glycosyltransferase, mucin, CHO, core saccharide

## Abstract

Sialylated glycans serve as key elements of receptors for many viruses, bacteria, and bacterial toxins. The microbial recognition and their binding specificity can be affected by the linkage of the terminal sugar residue, types of underlying sugar chains, and the nature of the entire glycoconjugate. Owing to the pathobiological significance of sialylated glycans, we have engineered Chinese hamster ovary (CHO) cells to secrete mucin-type immunoglobulin-fused proteins carrying terminal α2,3- or α2,6-linked sialic acid on defined *O*-glycan core saccharide chains. Besides stably expressing P-selectin glycoprotein ligand-1/mouse immunoglobulin G2b cDNA (PSGL-1/mIgG2b), CHO cells were stably transfected with plasmids encoding glycosyltransferases to synthesize core 2 (GCNT1), core 3 (B3GNT6), core 4 (GCNT1 and B3GNT6), or extended core 1 (B3GNT3) chains with or without the type 1 chain-encoding enzyme B3GALT5 and ST6GAL1. Western blot and liquid chromatography-mass spectrometry analysis confirmed the presence of core 1, 2, 3, 4, and extended core 1 chains carrying either type 1 (Galβ3GlcNAc) or type 2 (Galβ4GlcNAc) outer chains with or without α2,6-linked sialic acids. This panel of recombinant mucins carrying a repertoire of sialylated *O*-glycans will be important tools in studies aiming at determining the fine *O*-glycan binding specificity of sialic acid-specific microbial adhesins and mammalian lectins.

## 1. Introduction

*O*-GalNAc glycosylation of proteins contributes to protein structure and stability, and is a crucial element in protein-carbohydrate interactions [1]. It is a major form of glycosylation characterized by the covalent addition of glycans to the hydroxyl group of serine and threonine residues. In its predominant form, it is initiated by the addition of a GalNAc catalyzed by UDP-*N*-acetylgalactosamine: polypeptide *N*-acetylgalactosaminyltransferases (GALNTs) [1,2,3,4]. Elongation of this GalNAc generates eight different inner core structures, with core 1–4 being the most common ones [4,5]. The core structures can be further extended by the addition of other monosaccharide residues and terminally modified by sialylation, fucosylation, or sulfation [6,7]. Mucins, the main constituent of mucus, carry multiple *O*-GalNAc glycans that contribute to their highly extended and rigid structures. Cell surface-bound mucins that carry *O*-GalNAc glycans can block infection due to their ability to attract and bind to the carbohydrate binding receptors of various pathogens. *O*-GalNAc glycans on secreted mucins also function as a protective layer over the epithelium by saturating the bacterial adhesins, thereby masking the underlying host receptors [1].

A large number of microbial-host interactions are dependent on the recognition of specific sialylated ligands [8]. In most of these cases, the binding specificity can be modulated by many factors such as the types of sialic acid, their linkages to the underlying carbohydrate chain, the nature of the underlying monosaccharides, the presence or absence of concomitant fucosylation and/or sulfation, and the types of linkages (1,3 or 1,4) between inner monosaccharide moieties of the carbohydrate chain [9,10]. One of the most important determining factors for host specificity is the type of α-glycosidic linkage between sialic acids and the underlying oligosaccharide that defines the topology of the glycan. Glycans that contain 2,3-linked sialic acids exhibit a cone-like glycan topology, whereas the α2,6-linkage gives rise to an umbrella-like glycan topology that has greater flexibility and the potential to span a wider region of space [11]. One of the best-known interactions with sialylated glycans is the preferential binding of avian and human influenza virus to α2,3- and α2,6-linked sialic acids, respectively, a critical first step in the process of infection [12,13,14]. Oculotropic viruses like EV70 and adenovirus 37 (Ad37) also attach to α2,3-linked sialic acids [15]. However, the types of linkage are not sufficient to define the binding specificity of different strains because even dissimilarities between more distal carbohydrate residues can selectively be recognized by pathogens. For example, duck viruses have shown high affinity for receptors having a type 1 outer chain (lacto-*N*-biose or Galβ3GlcNAc) rather than a type 2 outer chain (*N*-acetyllactosamine or Galβ4GlcNAc) in the Neu5Acα2,3Gal-GlcNAc moieties [10]. Therefore, altering the receptor glycan structures by assigning them on various core chains can aid in defining the binding preference of various pathogens and improve the therapeutic design of putative viral inhibitors.

Mammalian lectin interactions with glycans have a critical function in cell-cell communication, pathogen binding, and immune responses. Mammalian lectins such as siglecs, selectins, and galectins show a broader diversity of specificity, covering most of the major glycan motifs [16]. Certain lectins are unique in their specificities for glycans and even a simple modification in the core structure can alter the specificity of the lectin [17]. Furthermore, the commercially available plant lectins and glycan-binding antibodies that are widely used as specific probes for glycan detection also differ in their glycan specificities within a particular specificity group. It is important to carefully characterize the specificities of these glycan-binding proteins. A reporter protein with a repertoire of target glycan structures harboring different linkages and substitutions would be useful.

Using the mucin-type fusion protein P-selectin glycoprotein ligand-1/mouse immunoglobulin G2b (PSGL-1/mIgG2b) as a probe, we have recently defined the *in vivo* specificity of *O*-glycan core chain glycosyltransferases transiently expressed in CHO-K1 cells [18]. PSGL-1/mIgG2b is produced as a dimer that carries 32 tandem mucin repeats, six potential *N*-glycosylation sites, and 106 potential *O*-glycosylation sites [19,20,21]. Even though the endogenous glycosylation machinery of CHO cells only supports the biosynthesis of mono- and di-sialylated core 1 *O*-glycan structures, it can be engineered to make core 2, core 3, and extended core 1 structures by the expression of the respective core enzymes [22,23,24]. CHO cells also lack the galactosyltransferase responsible for type 1 chain (Galβ3GlcNAc) biosynthesis. The biosynthesis of the type 1 chain is catalyzed by β1,3-galactosyltransferases (β3GalTs) that can transfer galactose to GlcNAc residues. Among the β3GalT family in humans, B3GALT1, B3GALT2, and B3GALT5 can make the type 1 chain on *N*-linked glycans, whereas only B3GALT5 was found to act on *O*-glycans [22]. In addition, we have shown that CHO cells have β1,4-galactosyltransferase (B4GALT) activity that can add β1,4-linked Gal to core 2 [25,26], core 3 [24], and extended core 1 [27] *O*-glycans, generating a type 2 outer chain (Galβ4GlcNAc). The recombinant proteins produced from CHO cells also exhibit a high degree of terminal α2,3 sialylation due to the endogenous expression of β-galactoside α2,3-sialyltransferase(s) (ST3GAL1–4) that catalyzes the addition of α2,3-linked sialic acids to galactoses [28,29,30,31]. CHO cells also express α-*N*-acetylgalactosaminide α2,6-sialyltransferase (ST6GALNAC4 and ST6GALNAC6), which was reflected in the *O*-glycans produced on our recombinant mucin-type fusion protein [31]. However, they lack β-galactoside α2,6-sialyltransferase (ST6GAL) activity that transfers sialic acids to the penultimate galactoses with an α2,6-linkage [28].

**Figure 1:**
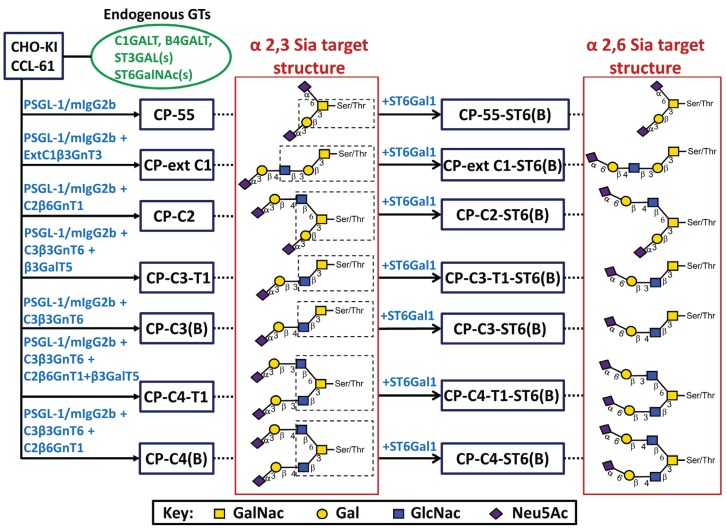
Stable CHO transfection scheme with designations of cell clones or bulk-selected cell populations and their respective target structures. CHO-K1 cells with their endogenous glycosylation machinery (green, oval box) were stably transfected with P-selectin glycoprotein ligand-1/mouse IgG2b Fc and *O*-glycan core chain glycosyltransferase cDNAs (blue) to generate stable CHO cell lines (blue boxes). In the name designation, C stands for CHO-K1, P for PSGL-1/mIgG2b, followed by the expected *O*-glycan core structures and (B) denotes bulk-selected cell populations. Based on the known specificity of the glycosyltransferases expressed, each cell line was expected to generate, among others, the indicated target structures (dotted line) based on different *O*-glycan core saccharide chains (dotted box). The *O*-glycan core structures were elongated by expressing B3GALT5 or by action of the endogenous B4GALTs to form type 1 or 2 outer chains, respectively. Terminal α2,3- or α2,6-sialylation was seen following the activity of a CHO-endogenous ST3GAL(s) or expression of β-galactoside α2,6-sialyltransferase 1 (ST6GAL1).

The aim of this study was to construct a repertoire of *O*-glycan core structures (core 1–4 and extended core 1) presenting terminal α2,3- and α2,6-linked sialic acid on type 1 or type 2 outer chains. Even though the cells are engineered to support the biosynthesis of certain sialylated *O*-glycan structures (Figure 1), the cells will generate an array of different *O*-glycan structures including alternative target structures and various non-sialylated precursor saccharides. Competition between endogenous and exogenously expressed glycosyltransferases for the same precursor chain may explain *O*-glycan heterogeneity. We believe that this panel of recombinant mucins carrying a repertoire of sialylated *O*-glycans will be an important tool of studies aimed at determining the fine *O*-glycan binding specificity of sialic acid-specific microbial adhesins and mammalian lectins.

## 2. Results and Discussion

### 2.1. Engineering of Stable CHO-K1 Cells Expressing PSGL-1/mIgG2b Carrying a Repertoire of Different Sialylated O-Glycans

The transfection scheme including the enzymes used to generate stable CHO cell lines secreting the mucin-type fusion protein PSGL-1/mIgG2b carrying core 1 (CP-55), extended core 1 (CP-ext C1), core 2 (CP-C2), core 3(CP-C3(B)), core 3 type 1 (CP-C3-T1), core 4 (CP-C4(B)), and core 4 type 1 (CP-C4-T1) *O*-glycans with and without terminal α2,6-linked sialic acids is shown in Figure 1. The CP-ext C1, CP-C2, CP-C3-T1, and CP-C4-T1 cells were expanded from individual cell clones handpicked by a PIPETMAN pipette following growth in medium containing selection drugs and were selected based on the concentration of fusion protein in the cell culture medium as assessed by enzyme-linked immunosorbent assay (ELISA). PSGL-1/mIgG2b produced in these clones were purified by Protein A affinity chromatography and gel filtration. The CP-C3(B), CP-C4(B) cells, and all the bulk-stable cell population expressing ST6GAL1 were selected in bulk and were, thus, not monoclonal. The fusion protein expressed in the bulk-selected cells was immunoaffinity-purified in batch using goat anti-mouse IgG agarose beads.

### 2.2. Expression and Characterization of PSGL-1/ mIgG2b Carrying the Major O-Glycan Core Structures

**Figure 2:**
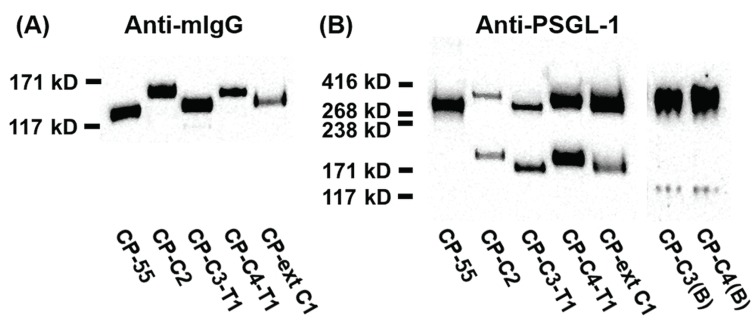
SDS-PAGE and Western blot analysis of purified PSGL-1/mIgG2b carrying α2,3-sialylated *O*-glycan core structures. For Western blot analyses, 0.2 μg of protein were loaded and analyzed on SDS-PAGE under reducing (**A**) or non-reducing (**B**) conditions. After blotting, membranes were probed with anti-mIgG Fc (**A**) and anti-PSGL-1 (**B**). The cell clone designation and how they were generated is shown in Figure 1.

SDS-PAGE and Western blot analyses of PSGL-1/mIgG2b expressed in the different cell lines revealed a protein of 100–180 kDa under reducing conditions (Figure 2A) and 250–350 kDa under non-reducing conditions (Figure 2B). Both anti-mouse IgG Fc and anti-PSGL-1 antibodies stain-purified PSGL-1/mIgG2b from all the stable clones (Figure 2). Expression of core 2 β1,6-*N*-acetylglucosaminyltransferase 1 (GCNT1) in CP-C2, CP-C4-T1, and CP-C4 can be verified by an increase in the size of PSGL-1/mIgG2b secreted from these cells compared to the fusion protein expressed in CP-55, and is explained by their more complex *O*-glycans (Figure 2A,B). Under reducing conditions, PSGL-1/mIgG2b purified from CP-C3-T1 and CP-ext C1 appeared to have a slightly increased molecular weight compared to PSGL-1/mIgG2b expressed in native CHO-K1 cells (Figure 2A).

**Figure 3:**
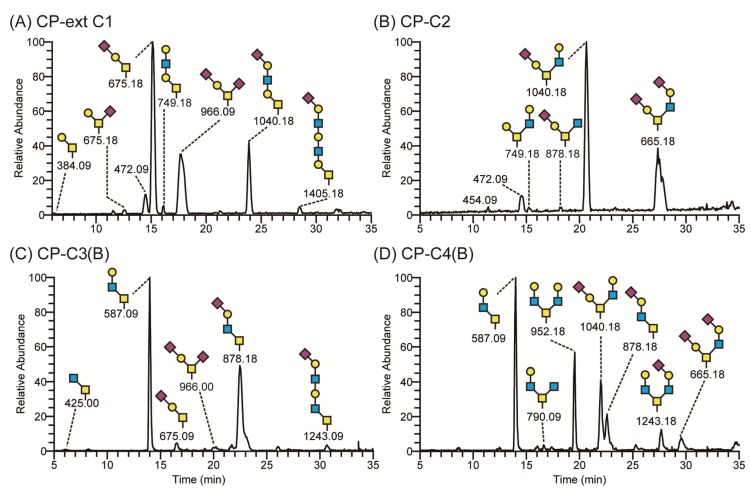
LC-MS chromatograms of *O*-glycans of recombinant PSGL-1/mIgG2b expressed in CHO-K1 cells stably transfected with *O*-glycan core glycosyltransferases. CHO-K1 cells stably expressing B3GNT3 and PSGL-1/mIgG2b (CP-ext C1) produced a fusion protein carrying sialylated core 1 and extended core 1 *O*-glycans (**A**); those expressing GCNT1 (CP-C2) had mostly core 2 *O*-glycans on the fusion protein (**B**); cells expressing B3GNT6 (CP-C3(B)) secreted PSGL-1/mIgG2b decorated with predominantly core 3 *O*-glycans (**C**); and cells expressing both GCNT1 and B3GNT6 (CP-C4(B)) carried mostly core 4 *O*-glycans on PSGL-1/mIgG2b (**D**). Proposed structures are depicted using the Consortium for Functional Glycomics symbol nomenclature.

The *O*-glycans of recombinant PSGL-1/mIgG2b produced in the different cell clones were analyzed by LC-MS (Figure 3). When CHO-K1 stably expressed the β1,3-*N*-acetylglucosaminyltransferase 3 (B3GNT3), 23.5% of the total *O*-glycans harbored the extended core 1 structure (Figure 3A and Table 1). Mono- and disialylated core 1 *O*-glycans were still the dominant structures (75.1% of total). As shown before in transient transfection experiments [18], core 3 *O*-glycans were also detected in cells stably transfected with B3GNT3. Expression levels were considerably lower in the stable clone (0.7% of total) compared to the cells transiently transfected with the enzyme [18]. We hypothesize that the B3GNT3 possess weak core 3 β1,3-*N*-acetylglucosaminyltransferase 6 (B3GNT6) activity as these enzymes show the highest homology among the β3-glycosyltransfease family members [32]. About 98% of the *O*-glycans contained the core 2 structure when GCNT1 was stably expressed in CHO-K1 cells, decreasing the level of core 1 *O*-glycans to 1.5% (Figure 3B and Table 1). When the core 3 enzyme, B3GNT6, was expressed stably (Figure 3C and Table 1), 86.2% of the *O*-glycans on CP-C3-expressed PSGL-1/mIgG2b contained core 3 *O*-glycans with Galβ4GlcNAcβ3GalNAcol (29.1%) and NeuAcα3Galβ4GlcNAcβ3GalNAcol (43.6%) being the predominant ones. The representation of core 3 structures among the *O*-glycans on the fusion protein expressed in the CP-C3(B) stable population was similar to that seen following transient expression (86%) [18]. Low amounts of core 2 *O*-glycans (1.6% of total) were also detected in this clone (Table 1), which we believe is due to the endogenous GCNT1 activity in CHO-K1 as described [18,33,34]. As expected, when both GCNT1 and B3GNT6 were expressed in CHO-K1, core 4 *O*-glycans were found on PSGL-1/mIgG2b (CP-C4(B), Figure 3D and Table 1). Since GCNT1 is able to convert core 1 and 3 to core 2 and 4, respectively, this stable cell line yielded *O*-glycans with core 1–4 (core 2, 36.8%; core 3, 30.5%; core 4, 31.2%). The distribution of core chains in this cell line suggests that the B3GNT6 converts core 1 to core 3 more efficiently than GCNT1 converts core 1 to core 2. To our surprise, four sulphated *O*-glycans (7.8%) were detected in this cell line (*m/z* 667, 1120, 1141, and 1641 in Table 1). In line with this, one sulphated *O*-glycan (1.2%; *m/z* 667 in Table 1) was also detected in the CP-C3(B) cell population. All sulphate groups were linked to carbon 6 of GlcNAc (6-SO_3_-GlcNAc) on both core 2 (*m/z* 667, 1120 and 1141) and core 4 (*m/z* 1641) *O*-glycans. This may suggest that the stable expression of B3GNT6 triggers expression or produces appropriate acceptors for endogenous GlcNAc 6-*O*-sulfotransferase(s) in CHO-K1.

**Table 1:** Putative *O*-glycan repertoires on PSGL-1/mIgG2b produced in *O*-glycan core chain-engineered CHO cells and identified by LC-MS/MS. The relative amounts of the different *O*-glycans are given in percentage (%) of the total sum of integrated peak areas in the LC-MS chromatograms. H, hexose; N, *N*-acetylhexosamine; S, sulphate; Na/Ng, NeuAc/NeuGc; B, bulk-selected cell populations.

Mass	Compsition		Putative structures	Core	exC1	C2	C3B	C4B	C3T1	C4T1	ST6B	exC1ST6B	C2ST6B	C3ST6B	C4ST6B	C3T1ST6B	C4T1ST6B
**384**	H1N1		Galβ1-3GalNAcol	1	0.5	—	—	—	2.0	—	5.2	6.6	5.5	—	—	—	3.9
**425**	N2		GlcNAcβ1-3GalNAcol	3	—	—	2.2	2.1	—	—	—	—	—	1.0	—	—	—
**587-1**	H1N2		Galβ1-3(GlcNAcβ1-6)GalNAcol	2	—	—	—	0.7	—	0.2	—	—	—	—	—	—	1.7
**587-2**	H1N2		Galβ1-4GlcNAcβ1-3GalNAcol	3	—	—	29.1	17.1	—	1.6	—	—	—	11.4	12.8	4.0	1.0
**587-3**	H1N2		Galβ1-3GlcNAcβ1-3GalNAcol	3	—	—	—	—	0.7	1.2	—	—	—	1.9	—	29.0	3.8
**667**	H1N2S1		Galβ1-4(6S)GlcNAcβ1-3GalNAcol	3	—	—	1.2	2.1	—	—	—	—	—	—	—	—	—
**675-1**	Na1H1N1		Galβ1-3(NeuAcα2-6)GalNAcol	1	1.0	—	1.2	0.2	1.9	—	4.1	3.6	2.3	—	—	—	1.1
**675-2**	Na1H1N1		NeuAcα2-3Galβ1-3GalNAcol	1	37.0	1.5	5.2	1.3	40.9	3.6	47.1	33.2	20.5	6.2	5.9	14.5	7.8
**691**	Ng1H1N1		NeuGcα2-3Galβ1-3GalNAcol	1	1.5	—	—	—	1.4	—	1.5	1.2	0.8	—	—	—	—
**749-1**	H2N2		Galβ1-3(Galβ1-4GlcNAcβ1-6)GalNAcol	2	—	1.3	1.3	1.3	—	2.5	—	—	1.8	—	2.7	—	8.2
**749-2**	H2N2		Galβ1-4GlcNAcβ1-3Galβ1-3GalNAcol	1	1.1	—	—	—	—	—	—	4.3	—	1.9	—	—	—
**790-1**	H1N3		GlcNAcβ1-3(Galβ1-4GlcNAcβ1-6)GalNAcol	4	—	—	—	0.5	—	0.1	—	—	—	—	2.8	—	3.2
**790-2**	H1N3		Galβ1-4GlcNAcβ1-3(GlcNAcβ1-6)GalNAcol	4	—	—	—	1.1	—	—	—	—	—	—	—	—	—
**829**	H2N2S1		Galβ1-3[Galβ1-4(6S)GlcNAcβ1-6]GalNAcol	2	—	—	—	—	—	0.3	—	—	—	—	—	—	2.1
**878-1**	Na1H1N2		NeuAcα2-6Galβ1-4GlcNAcβ1-3GalNAcol	3	—	—	—	—	—	—	—	—	—	0.7	—	—	—
**878-2**	Na1H1N2		NeuAcα2-3Galβ1-3(GlcNAcβ1-6)GalNAcol	2	—	1.7	—	0.8	—	2.8	—	—	—	3.0	7.3	—	1.8
**878-3**	Na1H1N2		NeuAcα2-3Galβ1-4GlcNAcβ1-3GalNAcol	3	0.7	—	43.6	7.6	—	1.7	—	—	—	20.3	—	—	—
**878-4**	Na1H1N2		NeuAcα2-3Galβ1-3GlcNAcβ1-3GalNAcol	3	—	—	—	—	1.7	—	—	—	—	—	11.4	39.7	1.9
**894**	Ng1H1N2		NeuGcα2-3Galβ1-3GlcNAcβ1-3GalNAcol	3	—	—	3.0	—	—	—	—	—	—	—	—	—	—
**952-1**	H2N3		Galβ1-4GlcNAcβ1-3(Galβ1-4GlcNAcβ1-6)GalNAcol	4	—	—	—	11.0	—	1.2	—	—	—	—	6.9	—	—
**952-1**	H2N3		Galβ1-4GlcNAcβ1-3Galβ1-4GlcNAcβ1-3GalNAcol	3	—	—	1.7	0.6	—	—	—	—	—	—	—	—	—
**966**	Na2H1N1		NeuAcα2-3Galβ1-3(NeuAcα2-6)GalNAcol	1	33.8	—	5.7	—	49.4	0.5	40.6	20.8	17.6	4.1	—	12.8	3.1
**982**	Na1Ng1H1N1		NeuGcα2-3Galβ1-3(NeuAcα2-6)GalNAcol	1	1.8	—	—	—	1.9	—	1.5	—	1.4	—	—	—	—
**1040-1**	Na1H2N2		NeuAcα2-6Galβ1-4GlcNAcβ1-3Galβ1-3GalNAcol	1	—	—	—	—	—	—	—	4.0	—	—	—	—	—
**1040-2**		Na1H2N2	NeuAcα2-3Galβ1-3(Galβ1-4GlcNAcβ1-6)GalNAcol	2	0.2	39.1	0.3	15.1	1.6	16.7	—	—	12.3	17.6	—	—	32.2
**1040-3**		Na1H2N2	NeuAcα2-3Galβ1-3(Galβ1-3GlcNAcβ1-6)GalNAcol	2	—	—	—	—	—	—	—	—	—	—	1.8	—	—
**1040-4**		Na1H2N2	Galβ1-3(NeuAcα2-3Galβ1-4GlcNAcβ1-6)GalNAcol	2	—	—	—	1.5	—	—	—	—	—	—	—	—	—
**1040-5**		Na1H2N2	NeuAcα2-3Galβ1-4GlcNAcβ1-3Galβ1-3GalNAcol	1	17.1	—	—	—	—	—	—	9.9	—	—	—	—	5.1
**1056**		Ng1H2N2	NeuGcα2-3Galβ1-4GlcNAcβ1-3Galβ1-3GalNAcol	1	1.0	—	—	—	—	—	—	—	1.1	—	—	—	—
**1081**		Na1H1N3	GlcNAcβ1-3(NeuAcα2-3Galβ1-4GlcNAcβ1-6)GalNAcol	4	—	—	—	1.1	—	—	—	—	—	—	—	—	—
**1114**		H3N3	Galβ1-4GlcNAcβ1-3Galβ1-4GlcNAcβ1-3Galβ1-3GalNAcol	1	0.4	—	—	—	—	—	—	4.5	—	—	—	—	—
**1120-1**		Na1H2N2S1	NeuAcα2-3Galβ1-3[Galβ1-4(6S)GlcNAcβ1-6]GalNAcol	2	—	—	—	1.2	—	6.8	—	—	4.5	7.2	—	—	—
**1120-2**		Na1H2N2S1	NeuAcα2-3Galβ1-4(6S)GlcNAcβ1-3Galβ1-3GalNAcol	1	—	—	—	—	—	—	—	—	—	—	2.9	—	1.2
**1243-1**		Na1H2N3	NeuAcα2-3Galβ1-4GlcNAcβ1-3(Galβ1-4GlcNAcβ1-6)GalNAcol	4	—	—	—	2.1	—	—	—	—	—	—	—	—	—
**1243-2**		Na1H2N3	Galβ1-4GlcNAcβ1-3(NeuAcα2-3Galβ1-4GlcNAcβ1-6)GalNAcol	4	—	—	—	6.5	—	0.7	—	—	—	—	—	—	—
**1243-3**		Na1H2N3	NeuAcα2-3Galβ1-4GlcNAcβ1-3Galβ1-4GlcNAcβ1-3GalNAcol	3	—	—	4.7	1.1	—	—	—	—	—	—	—	—	—
**1331-1**		Na2H2N2	NeuAcα2-3Galβ1-3(NeuAcα2-6Galβ1-4GlcNAcβ1-6)GalNAcol	2	—	—	—	—	—	—	—	—	14.3	13.7	—	—	—
**1331-2**		Na2H2N2	NeuAcα2-3Galβ1-3(NeuAcα2-3Galβ1-4GlcNAcβ1-6)GalNAcol	2	—	54.4	—	8.6	0.5	53.8	—	—	—	—	45.5	—	18.0
**1347**		Na1Ng1H2N2	NeuGcα2-3Galβ1-3(NeuAcα2-3Galβ1-4GlcNAcβ1-6)GalNAcol	2	—	—	—	1.9	—	1.3	—	—	—	—	—	—	—
**1405-1**		Na1H3N3	NeuAcα2-6Galβ1-4GlcNAcβ1-3Galβ1-4GlcNAcβ1-3Galβ1-3GalNAcol	1	—	—	—	—	—	—	—	—	—	3.9	—	—	2.1
**1405-2**		Na1H3N3	NeuAcα2-3Galβ1-3(Galβ1-4GlcNAcβ1-3Galβ1-4GlcNAcβ1-6)GalNAcol	2	—	0.7	—	2.2	—	0.8	—	—	—	—	—	—	—
**1405-3**		Na1H3N3	NeuAcα2-3Galβ1-4GlcNAcβ1-3Galβ1-4GlcNAcβ1-3Galβ1-3GalNAcol	1	3.4	—	—	—	—	—	—	11.9	—	—	—	—	—
**1411**		Na2H2N2S1	NeuAcα2-3Galβ1-3[NeuAcα2-3Galβ1-4(6S)GlcNAcβ1-6]GalNAcol	2	—	—	—	3.5	—	3.7	—	—	13.3	—	—	—	—
**1534**		Na2H2N3	NeuAcα2-3Galβ1-4GlcNAcβ1-3(NeuAcα2-3Galβ1-4GlcNAcβ1-6)GalNAcol	4	—	—	—	6.2	—	—	—	—	—	—	—	—	—
**1550**		Na1Ng1H2N3	NeuAcα2-3Galβ1-4GlcNAcβ1-3(NeuAGcα2-3Galβ1-4GlcNAcβ1-6)GalNAcol	4	—	—	—	1.0	—	—	—	—	—	—	—	—	—
**1608**		Na1H3N4	NeuAcα2-3Galβ1-4GlcNAcβ1-3Galβ1-4GlcNAcβ1-3Galβ1-4GlcNAcβ1-3GalNAcol	1	—	—	0.7	—	—	—	—	—	—	—	—	—	—
**1614**		Na2H2N3S1	NeuAcα2-3Galβ1-4GlcNAcβ1-3[NeuAGcα2-3Galβ1-4(6S)GlcNAcβ1-6]GalNAcol	3	—	—	—	1.0	—	—	—	—	—	—	—	—	—
**1696-1**		Na2H3N3	NeuAcα2-3Galβ1-3(NeuAcα2-6Galβ1-4GlcNAcβ1-3Galβ1-4GlcNAcβ1-6)GalNAcol	2	—	—	—	—	—	—	—	—	4.6	7.1	—	—	—
**1696-2**		Na2H3N3	NeuAcα2-3Galβ1-3(NeuAcα2-3Galβ1-4GlcNAcβ1-3Galβ1-4GlcNAcβ1-6)GalNAcol	3	—	1.2	—	—	—	0.5	—	—	—	—	—	—	1.8
**1771**		Na1H4N4	NeuAcα2-3Galβ1-4GlcNAcβ1-3Galβ1-4GlcNAcβ1-3Galβ1-4GlcNAcβ1-3Galβ1-3GalNAcol	3	0.6	—	—	—	—	—	—	—	—	—	—	—	—
**1899**		Na2H3N4	NeuAcα2-3Galβ1-4GlcNAcβ1-3(NeuAcα2-3Galβ1-4GlcNAcβ1-3Galβ1-4GlcNAcβ1-6)GalNAcol	3	—	—	—	0.7	—	—	—	—	—	—	—	—	—

### 2.3. Expression and Characterization of PSGL-1/mIgG2b Carrying O-Glycans Extended with Type 1 Outer Core Chains

**Figure 4:**
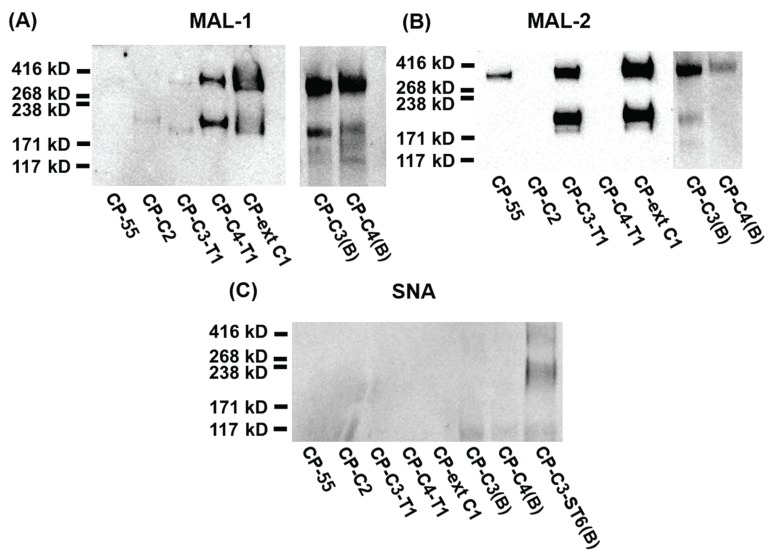
SDS-PAGE and Western blot analysis of purified PSGL-1/mIgG2b carrying α2,3-sialylated *O*-glycan core structures. For Western blot analyses, 0.2 μg of recombinant protein were loaded and analyzed on SDS-PAGE under non-reducing conditions. After blotting, membranes were probed with *Maackia amurensis* lectin-1 (MAL-1) (**A**), *Maackia amurensis* lectin-2 (MAL-2) (**B**), and *Sambucus nigra* agglutinin (SNA) (**C**). MAL-1 recognizes the type 2 chain (Galβ4GlcNAc) with or without α2,3-linked sialic acid; MAL-2 detects terminal α2,3-linked sialic acids; SNA detects terminal α2,6-linked sialic acids.

We introduced the human B3GALT5 into CHO-K1 together with the core 3 or 4 *O*-glycan synthesizing enzymes in order to engineer core 3 or 4 *O*-glycans with a type 1 (Galβ3GlcNAc) outer core chain [6,35]. *O*-glycans with type 1 or 2 outer core chains were analyzed by lectin Western blotting using *Maackia amurensis* lectin-1 (MAL-1, Figure 4A). MAL-1 recognizes the type 2 chain (Galβ4GlcNAc) with or without α2,3-linked sialic acid. PSGL-1/mIgG2b produced in CP-ext C1, CP-C3(B), and CP-C4(B) reacted strongly with MAL-1 (Figure 4A), suggesting the presence of the type 2 chain on these core structures, an interpretation also supported by the LC-MS analyses (Figure 3A, C and D). The fusion protein produced in CP-C3-T1, which carries the type 1 chain on core 3 *O*-glycans, stained very weakly with MAL-1 (Figure 4A, lane 3), indicating that MAL-1 lectin requires the type 2 chain for optimal binding. The weak MAL-1 staining also suggests that most of the core 3 structures produced in the CP-C3-T1 clone are elongated with the type 1 chain. This appears not to be the case for the CP-C4-T1 clone because PSGL-1/mIgG2b produced in this clone stained strongly with MAL-1 (Figure 4A). Even though we have stably expressed the type 1 chain-generating enzyme B3GALT5 in the CP-C3-T1 and CP-C4-T1 clones, type 2 chain *O*-glycans can still be present on the fusion protein because of the competition between B3GALT5 and the endogenous B4GALT(s) enzyme(s). Although there is one glycan with the type 2 chain on the core 2 structures detected on the fusion protein produced in CP-C2, MAL-1 staining was very weak, suggesting core-chain dependent recognition of the NeuAcα3Galβ4GlcNAc determinant by MAL-1 [18]. This also suggests that the binding of MAL-1 to fusion proteins from CP-C3-T1 and CP-C4-T1 reflects the amount of type 2 chain on the C3 branch rather than on the C6 branch, which was also confirmed by LC-MS/MS (see following section and Table 1). Mono- and disialylated core1 *O*-glycans produced by the fusion protein from stable clone CP55 [36] were negative for MAL-1 staining.

**Figure 5:**
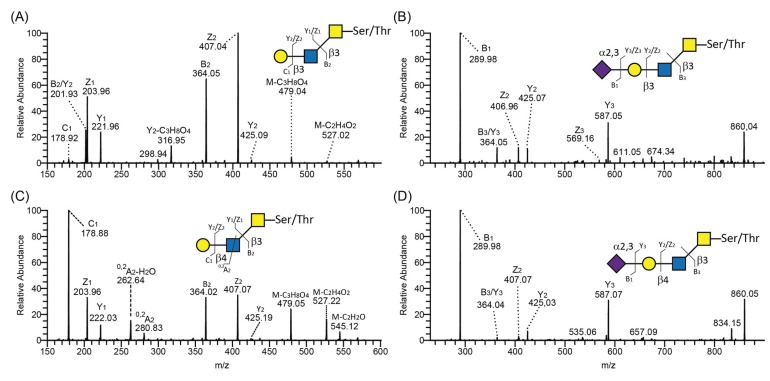
LC-MS/MS spectra of core 3 *O*-glycans containing type 1 and 2 outer core chains. MS/MS spectra of type 1 (**A**,**B**) and type 2 (**C**,**D**) chain-containing *O*-glycans on core 3 released from PSGL-1/mIgG2b produced in CP-C3-T1. Proposed structures are depicted using the Consortium for Functional Glycomics symbol nomenclature. A schematic characteristic glycosidic or cross-ring cleavage is shown according to the Domon and Costello nomenclature [37].

Using negative-ion mode LC-MS/MS, type 2 and 1 chains were identified by the presence or absence, respectively, of diagnostic ions arising from a cross-ring cleavage of GlcNAc (^0,2^A_GlcNAc_-H_2_O and ^0,2^A_GlcNAc_) [38]. For example, both type 1 (Figure 5A) and type 2 (Figure 5C) chains were found on core 3 *O*-glycans derived from PSGL-1/mIgG2b produced in CP-C4-T1. The presence of cross-ring cleavage ions at *m/z* 281 (^0,2^A_2_) and 263 (^0,2^A_2_-H_2_O) (Figure 5C) leads us to assign this structure as a core 3 *O*-glycan with type 2 chain extension Galβ4GlcNAcβ3GalNAcol. In contrast to the type 2 chain, the type 1 chain usually lacks these fragmentation ions, especially the fragmentation ions at ^0,2^A_GlcNAc_-H_2_O (*i.e.*, missing the fragmentation ions at *m/z* 263 in Figure 5A). In addition, isomeric *O*-glycans carrying either type 1 or 2 chains differ with regard to their retention time on the graphitized carbon column. In general, the type 2 chain-containing *O*-glycans elute earlier on the column. The CP-C4-T1-derived type 2 chain on core 3 *O*-glycan eluted at 13.26 min (587-2 in Table 1) while the type 1 chain on core 3 eluted at 13.70 min (587-3 in Table 1). Unlike neutral glycans, sialylated type 1 and 2 glycans showed almost identical MS/MS spectra as exemplified by the sialylated type 1 chain on core 3 (Figure 5B) and the sialylated type 2 chain on core 3 *O*-glycans (Figure 5D) derived from PSGL-1/mIgG2b produced in CP-C3-T1 (878-4 in Table 1) and CP-C4-T1 (878-3 in Table 1). The only difference was that the sialylated type 2 chain eluted earlier than the sialylated type 1 chain-containing glycans. Thus, annotating sialylated *O*-glycans differing only with regard to the outer core saccharide chain, *i.e.*, type 1 or 2, can be difficult and caution should be taken not to mix them up.

The *O*-glycans of PSGL-1/mIgG2b produced in CP-C3-T1 contained only two glycans carrying the type 1 outer chain and accounting for 2.4% of total *O*-glycans (587-3 and 878-4 in Table 1). The majority was mono- or disialylated core 1 *O*-glycans (95.5% of total *O*-glycan), which are devoid of type 1 and 2 outer chains. As was the case for the *O*-glycan repertoire carried by the fusion protein expressed in CP-C3(B), core 2 *O*-glycans were poorly (2.1% of total) represented among the *O*-glycans on PSGL-1/mIgG2b produced in CP-C3-T1. The only glycan containing the type 2 chain was the monosialylated core 2 structure NeuAcα3Galβ3(Galβ4GlcNAcβ6)GalNAcol (1.6%; 1040-2 in Table 1). This explains the weak binding of the lectin MAL-1 to CP-C3-T1-produced PSGL-1/mIgG2b. The high amount of core 1 *O*-glycans in this clone is likely to be an effect of clonal selection because, in the case of bulk-selected ST6GAL1 stable CHO cells, the amount of core 1 *O*-glycans was not as high as in these colony-selected cell clones (CP-C3-T1-ST6(B), 28.2%; CP-C4-T1-ST6(B), 22.2%).

The fusion protein produced in CP-C4-T1 stained strongly with MAL-1, indicating the presence of type 2 chain *O*-glycans (Figure 4A). MAL-1 binds poorly to the type 2 chain on the core 2 *O*-glycan or C6 branch of GalNAc, whereas the strong MAL-1 staining of the fusion protein produced in CP-C4-T1 is due to the type 2 chain on the C3 branch of GalNAcol. LC-MS analysis confirmed a high representation of the type 2 chain (94.7%, Table 1) on both C3 and C6 branches of *O*-glycans released from the fusion protein produced in CP-C4-T1, the most abundant one being a core 2 *O*-glycan with a type 2 outer core saccharide chain. Only one glycan with the type 1 chain on the C3 branch was detected on the fusion protein expressed in CP-C4-T1 (1.2%; 587-3 in Table 1 and Figure 5A). Stable expression of B3GALT5 in CP-C4-T1 also switched the ratio of the core structures such that the representation of core 2 chains increased from 36.8% to 89.4%; core 3 decreased from 30.5% to 4.6%; and core 4 dropped from 31.2% to 2.0%. This difference in the distribution of *O*-glycans between type 1 and type 2 in core 3/core 4 clones can be due to clone-specific events as the type 1 clones are monoclonal whereas the type 2 clones are bulk-selected. However, similar to CP-C4(B), three sulphated *O*-glycans were also detected in CP-C4-T1 (10.8% in total; 829, 1120-2, and 1411 in Table 1).

### 2.4. Expression and Characterization of PSGL-1/ mIgG2b Carrying α2,3- and α2,6-Sialylated O-Glycan Core Structures

We used MAL-2 and *Sambucus nigra* agglutinin (SNA) to detect terminal α2,3- and α2,6-linked sialic acids, respectively. MAL-2 stained the fusion protein produced in CP-55 (Figure 4B), indicating high levels of α2,3-linked sialic acid. This was also true for the fusion protein produced in CP-ext C1 (Figure 4B), which gave a much stronger signal upon MAL-2 staining, suggesting that it carries more terminal α2,3-linked sialic acid compared to the fusion protein from CP-55. PSGL-1/mIgG2b produced in CP-C3-T1 and CP-C3(B) (Figure 4B) were both positive for MAL-2 staining, whereas the CP-C2 and CP-C4-T1 fusion proteins (Figure 4B) were negative despite having α2,3-linked NeuAc. Based on the LC-MS/MS analysis, 89.4% of the *O*-glycans from PSGL-1/mIgG2b produced in CP-C4-T1 were based on the core 2 *O*-glycan (Table 1). Only 1.7% of all CP-C4-T1 *O*-glycans contained α2,3-linked NeuAc on a core 3 chain. Thus, we believe that the poor reactivity of MAL-2 with PSGL-1/mIgG2b expressed in CP-C2 and CP-C4-T1 is explained by a core chain dependence of its binding and a relatively lower affinity for core 2 and 4 *O*-glycans despite abundant α2,3-sialylation. Also PSGL-1/mIgG2b expressed in CP-C4(B) stained very weakly with MAL-2 (Figure 4B), which may be explained by a low representation of sialic acid on core 3 chains (8.7%) among the *O*-glycans released from this fusion protein (Table 1).

**Figure 6:**
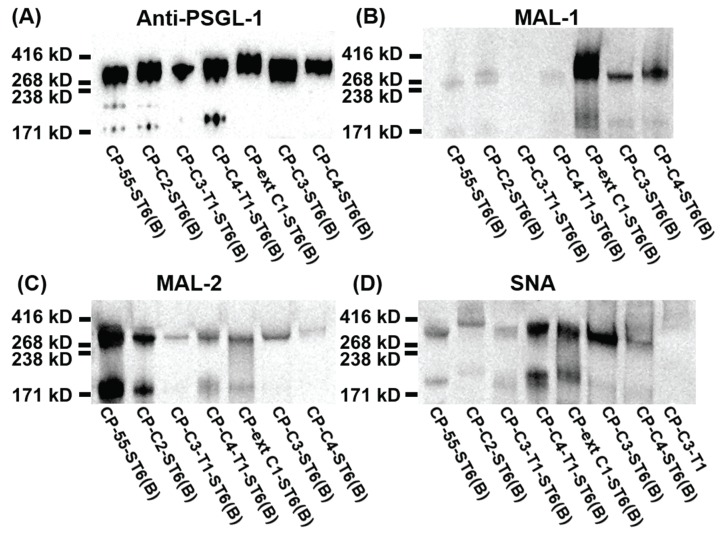
SDS-PAGE and lectin blot analysis of purified PSGL-1/mIgG2b carrying α2,6-linked sialic acid on different *O*-glycan core structures. For Western blot analyses, 0.2 μg of recombinant protein were loaded and analyzed on SDS-PAGE under non-reducing conditions. After blotting, membranes were probed with anti-PSGL-1 (**A**), MAL-1 (**B**), MAL-2 (**C**), and SNA (**D**).

SNA staining was used to detect α2,6-linked sialic acid on purified PSGL-1/mIgG2b produced in the bulk stable CHO cell population expressing human ST6GAL1. All the stable clones lacking ST6GAL1 were negative upon SNA staining, confirming that CHO cells only have ST3GALT activity [28] (Figure 4C and Table 1). PSGL-1/mIgG2b produced in all the ST6GAL1 stable cell populations had an apparent molecular weight of 250–350 kDa upon staining with the anti-PSGL-1 antibody under non-reducing conditions (Figure 6A). When probing the membranes with MAL-1, PSGL-1/mIgG2b produced in CP-ext C1-ST6(B), CP-C3-ST6(B), and CP-C4-ST6(B) stained strongly, indicating consistent expression of type 2 chains on these proteins (Figure 6B). However, the fusion proteins produced in CP-55-ST6(B), CP-C2-ST6(B), CP-C3-T1-ST6(B), and CP-C4-T1-ST6(B) (Figure 6B, lanes 1–4) were negative due to the core chain dependent activity of MAL-1 and lack (or low amounts) of type 2 chains on the appropriate core structures (Table 1). MAL-2 stained PSGL-1/mIgG2b produced in CP-55-ST6(B) and CP-C2-ST6(B) strongly (Figure 6C) and those expressed in the other ST6GALT1 stable populations weakly. Even though MAL-2 does not bind α2,3-linked sialic acid on a core 2 structure, the binding to PSGL-1/mIgG2b produced in CP-C2-ST6(B) can be explained by the presence of sialylated core 1 *O*-glycans on this fusion protein as revealed by the LC-MS analysis (Table 1). Strong SNA binding was observed to PSGL-1/mIgG2b produced in CP-C4-T1-ST6(B), CP-ext C1-ST6(B), and CP-C3-ST6(B) (Figure 6D) and a weaker binding of the other clones (Figure 6D). This can be either due to the lectin specificity or a core chain preference of ST6GAL1. LC-MS/MS analysis of these clones suggested the discrepancy was caused by lectin specificity against different core chains (see following section).

To define the *O*-glycan profile after stable expression of ST6GAL1, LC-MS/MS analysis was performed. The presence of α2,6-sialylated *O*-glycans was determined by two criteria: (1) the presence of specific fragmentation ions of α2,6-linked sialic acid in the MS/MS spectra [38]; and (2) shorter retention times on the graphitized carbon column [18].

When ST6GAL1 was co-expressed with PSGL-1/mIgG2b alone (CP-55-ST6), no *O*-glycans with terminal α2,6-linked sialic acids were detected besides the disialyl core 1 structure (675-1 and 966 in Table 1). This result is consistent with the lectin staining using MAL-1 and -2. Weak binding to SNA is probably due to the α2,6-sialylation of *N*-glycans on PSGL-1, which was also observed in our previous study in which ST6GAL1 was transiently expressed [18].

When ST6GAL1 was stably expressed in CP-ext C1 (CP-ext C1-ST6(B)), one *O*-glycan based on extended core 1 and with an α2,6-linked NeuAc (1040-1 in Table 1) was detected. Compared to the representation of α2,3-sialylated extended core 1 (21.8%), the representation of α2,6-sialylated extended core 1 (4.0%) was low. Like the fusion protein produced in CP-ext C1, the major *O*-glycans of PSGL-1/mIgG2b expressed in CP-ext C1-ST6 were non-extended core 1 (65.4% *vs.* 75.6%), including mono- (675-1, 675-2, and 691 in Table 1) and disialylated core 1 (966 and 982 in Table 1). The LC-MS/MS data of PSGL-1/mIgG2b expressed in CP-ext C1-ST6(B) were consistent with the results of the lectin blots, in which this fusion protein was reactive with all lectins tested (SNA, MAL-1, and -2).

In case of PSGL-1/mIgG2b produced in CP-C2-ST6(B), LC-MS/MS analysis revealed 50.8% of total glycans were core 2 *O*-glycans, and only one structure with α2,6-linked sialic acid was detected (NeuAcα3Galβ3(NeuAcα6Galβ4GlcNAcβ6)GalNAcol; 14.3% of total; 1331-1 in Table 1). We could only detect a weak SNA binding to PSGL-1/mIgG2b produced in CP-C2-ST6(B), which can be due to the core chain-dependent activity of SNA [18]. Sialylated core 1 *O*-glycans were also detected on the fusion protein produced in CP-C2-ST6(B) as revealed by the LC-MS analysis, which may explain the MAL-2 staining of the fusion protein (Figure 6C). Two sulphated core 2 *O*-glycans (1120-1 and 1411 in Table 1) were identified, constituting 17.8% of all *O*-glycans on this fusion protein.

No α2,6-sialylation was detected on *O*-glycans of PSGL-1/mIgG2b expressed in CP-C3-T1-ST6(B), while four α2,6-sialylated *O*-glycans were found on the fusion protein produced in CP-C3-ST6(B) (878-1, 1331-1, 1405-1, and 1696-1 in Table 1). Human ST6GAL1 has high activity against type 2 outer chains, but it cannot use type 1 outer chains (Galβ3GlcNAc) or core 1 *O*-glycans (Galβ3GalNAcol) [39]. Our LC-MS/MS results confirmed that human ST6GAL1 cannot use the type 1 outer chain as an acceptor. Galβ3GlcNAcβ3GalNAcol (587-3 in Table 1) and NeuAcα3Galβ3GlcNAcβ3GalNAcol (878-1 in Table 1) were the two major *O*-glycan structures (68.7% of all *O*-glycans) on PSGL-1/mIgG2b produced in CP-C3-T1-ST6(B). This is consistent with the weak reactivity of this fusion protein with MAL-1 and MAL-2 (Figure 6B,C).

LC-MS/MS revealed that core 1-4 *O*-glycans were present on PSGL-1/mIgG2b produced in both CP-C4-T1-ST6(B) and CP-C4-ST6(B). *O*-glycans based on core 2 were predominant on both proteins (65.8% for CP-C4-T1-ST6(B) and 57.3% for CP-C4-ST6(B)). In contrast, core 4-containing *O*-glycans were poorly represented on both proteins (3.2% for CP-C4-T1-ST6 and 9.7% for CP-C4-ST6). Like the fusion protein produced in CP-C3-T1-ST6(B), no α2,6-sialylated type 1 chains were detected on PSGL-1/mIgG2b produced in CP-C4-T1-ST6(B). Two type 1 chain-containing *O*-glycans were detected on core 3 *O*-glycans (587-3 and 878-1, Table 1). Only one α2,6-sialyled *O*-glycan was detected on the fusion protein produced in CP-C4-T1-ST6(B), and it was shown to be based on an extended core 1 *O*-glycan (2.1%, 1405-1 in Table 1) most likely generated by the B3GNT6. This explains why the fusion protein expressed in CP-C4-T1-ST6(B) binds to SNA (Figure 6D) and confirms that human ST6GAL1 cannot utilize the type 1 chain as an acceptor. However, PSGL-1/mIgG2b expressed in CP-C4-ST6(B) did not carry any detectable α2,6-sialylated *O*-glycans as revealed by LC-MS/MS. This is consistent with the weak binding (if any) to SNA (Figure 6D). Because we can detect α2,6-linked sialic acid on PSGL-1/mIgG2b expressed in CP-C4-T1-ST6(B), we believe the lack of α2,6-linked sialic acid on the fusion protein produced in CP-C4-ST6(B) is due to the relatively low amount of fusion protein analyzed. The presence of type 2 chains and α2,3-linked sialic acid on fusion proteins produced in CP-C4-ST6(B) and CP-C4-T1-ST6(B) was consistent with the binding reactivity with MAL-1 and -2 (Figure 6B,C).

## 3. Experimental Section

### 3.1. Cell Culture and Expression Vectors

The CHO-K1 cell line (ATCC, Manassas, VA, USA) or stably transfected CHO-K1 cell lines were cultured in Dulbecco’s modified Eagle’s medium (DMEM) (Lonza Group Ltd., Basel, Switzerland) supplemented with 10% fetal bovine serum (FBS) (Invitrogen), 2 mM L-glutamine (Invitrogen), 100 units/mL penicillin, and 100 μg/mL streptomycin (Invitrogen). Cells were maintained in a humidified incubator at 37 °C and 5.0% CO_2_.

The CHO-K1 cell line adapted to serum-free conditions was cultured in ProCHO-4 (Lonza Group Ltd.) medium supplemented with 2 mM L-glutamine, 100 µg/mL dextran sulphate (Sigma-Aldrich, St Louis, MO, USA), and 25 µg/mL gentamicin. Cells were maintained as single-cell suspension cultures in shaker flasks (Corning Inc., Corning, NY, USA) at 100 rpm, 37 °C, and 5.0% CO_2_.

The information about the expression vectors used for the stable cell line engineering is given in Table 2. Prior to transfection, all expression vectors were linearized with *Avr*II (New England BioLabs, Ipswich, MA, USA) except ST6GAL1 which was linearized with *Spe*I (New England BioLabs).

The concentration of the selection drug, based on the antibiotic cassette of each transfected expression vector (Table 2), in the medium were as follows: puromycin 4 μg/mL (Invitrogen), geneticin 400 μg/mL (G418 sulfate, Invitrogen), zeocin 50 μg/mL (Invitrogen), blasticidin 2 μg/mL (Invitrogen), hygromycin 200 μg/mL (Invitrogen), and GPT selection reagent (25 μg/mL mycophenolic acid, 0.25 μg/mL xanthine, 13.6 μg/mL hypoxanthine, Sigma-Aldrich).

**Table 2:** Expression vectors used in this study.

Expression vector	cDNA	Resistance gene	Source	Reference
EF1α/PSGL-1/EK/mIgG2b	PSGL-1/mIgG2b fusion gene	Puromycin acetyltransferase (puromycin resistance)	HL-60 cDNA library	[36]
EF1α/C2 β6GnT1	β1,6-*N*-acetylglucosaminyltransferase 1 (GCNT1)	Neomycin phosphotransferase (G418 resistance)	HL-60 cDNA library	[25]
CMV/C3 β3GnT6	β1,3-*N*-acetylglucosaminyltransferase 6 (B3GNT6)	Hygromycin resistance	Human stomach cDNA library	[23]
CMV/β3GalT5	β1,3-galactosyltransferase 5 (B3GALT5)	Guanosine phosphoribosyl transferase (mycophenolic acid, xanthine and hypoxanthine resistance)	Human placental cDNA library	[23]
CMV/extended C1 β3GnT3	β1,3-*N*-acetylglucosaminyltransferase 3 (B3GNT3)	Sh*Ble* (zeocin) resistance	HT-29 cDNA library	[18]
CMV/ST6Gal 1	β-galactoside α 2,6 sialyltransferase 1 (ST6GAL1)	Blasticidin S deaminase (blasticidin resistance	Human placental cDNA library	[18]

### 3.2. Transfection and Clonal Selection of Glyco-Engineered CHO Cells

CHO-K1 cells were seeded in a 75 cm^2^ tissue culture flask on the day before transfection. Adherently growing cells at 90%–95% confluence were transfected with the linearized expression vectors using Lipofectamine 2000™ transfection reagent (Invitrogen) according to the manufacturer’s instructions. The repertoire of stable CHO transfectants was generated by simultaneous transfection of multiple expression vectors as described in the transfection scheme (Figure 1 and Table 2). Twenty-four hours following transfection, the cells were split into five 100-mm tissue culture dishes and incubated in medium containing the respective selection reagents as guided by the plasmid used and starting 48 h after transfection. The concentrations of the selection drugs are given in Section 3.1. The selection medium was changed every second-to-third day. After approximately two weeks drug resistant clones were identified as colonies under the microscope. In case of CP-C2, CP-C3-T1, CP-C4-T1, and CP-ext C1, single clones were hand-picked using sterile pipette tips and transferred to 96-well plates in selection medium. Sixty clones from each stable transfection were screened in the 96-well format for expression of PSGL-1/mIgG2b in supernatants by a sandwich ELISA method as described previously [40]. Twelve clones were further expanded and analyzed for the expression of PSGL-1/mIgG2b by SDS-PAGE and Western blotting. Due to the lack of a suitable lectin or antibody to detect the *O*-glycan core structures, we selected the high-expressing clones based on the expression of the recombinant fusion protein. The selected six stable CHO clones were sequentially adapted to serum-free culture. The production level of PSGL-1/mIgG2b in these clones was further assessed by ELISA. The high-producing clones of CP-C2, CP-C3-T1, CP-C4-T1, and CP-ext C1 had a yield of 1.4, 2.1, 2.5, and 1.7 μg/mL, respectively. These clones were subsequently transferred to shaker flasks for large-scale cultivation.

CP-C3(B), CP-C4(B), and all the α2,6-sialylated stable clones were bulk-selected and the resistant colonies in the dish were all collected by trypsinization, pooled, and were further expanded in 75 cm^2^ tissue culture flasks. The concentrations of recombinant fusion protein in the supernatants of bulk-selected stable clones were quantified using ELISA in the range of 150–300 ng/mL.

### 3.3. Production and Purification of PSGL-1/mIgG2b Produced in Glyco-Engineered CHO Cells

High-producing single-cell clones of CP-C2, CP-C3-T1, CP-C4-T1, and CP-ext C1 were cultured in serum-free ProCHO-4 medium in 3 L shaker flasks as described in the Section 3.1. At regular intervals, fresh cultivation medium was added to a cell concentration of 1.5 × 10^5^ cells/mL until the final volume of 1.5 L was reached in the shaker flasks. The culture was harvested when the final cell density had reached 3–4 × 10^6^ cells/mL and the viability had dropped to 70%. The cell culture supernatant was centrifuged at 5020× *g* for 30 min to remove the cell debris and was further clarified by filtration using a 0.45 μm vacuum-driven filtration system (Millipore, Billerica, MA, USA).

All chromatographic procedures were carried out on an ÄKTAExplorer 100 controlled by Unicorn software (v. 5.11) (GE Healthcare, Amersham, Uppsala, Sweden). The clarified supernatants were sterile filtered with 0.22 µm polyether sulfone filter (Nalgene) before loading onto a MabSelect SuRe column (GE Healthcare) pre-equilibrated with phosphate buffered saline (PBS). The column was washed with 10 column volumes (CV) of PBS, and elution of recombinant fusion protein was achieved using 5 CV of 0.1 M sodium citrate, pH 3.0. After elution, selected fractions were pooled, neutralized with 300 μL per mL of 1 M Tris-HCl, pH 9.0 and then dialyzed extensively (12–14 kDa cut-off) against MilliQ water at 4 °C. After dialysis, the samples were frozen, lyophilized, and stored at −80 °C before further purification.

Lyophilized samples were dissolved to approximately 5 mg/mL in gel filtration buffer (0.1 M sodium phosphate, pH 7.2, 0.5 M NaCl). Gel filtration of the PSGL-1/mIgG2b was carried out on a pre-equilibrated HiPrep 26/60 Sephacryl S-300 HR column (26 × 60 mm, GE Healthcare). Typically, 5 mL sample was applied to the gel filtration column and eluted with a flow rate of 1 mL/min. Eluted fractions were kept at 4 °C until pooling were done on the basis of Western blot analysis. Pooled fractions were then dialyzed, frozen, lyophilized, and stored at −80 °C.

The bulk-selected stable cell population generated for expressing recombinant PSGL-1/mIgG2b fusion proteins carrying CP-C3(B), CP-C4(B), and α2,6 sialylated *O*-glycans were cultured and expanded in 75 cm^2^ tissue culture flask containing DMEM media with selection drugs and 1.5% FBS. The cell culture supernatant was collected after eight days and was centrifuged at 5020× *g* for 30 min to remove the cell debris. The different recombinant PSGL-1/mIgG2b fusion proteins were then purified from 100 mL clarified supernatant using goat anti-mouse IgG agarose beads (50 μL slurry per 10 mL supernatant, Sigma-Aldrich) by rolling head over tail at 4 °C overnight. The beads with fusion proteins were washed three times in PBS and subjected to SDS-PAGE electrophoresis, Western blot, and *O*-glycan analysis. The concentrations of recombinant fusion protein in supernatants and in purified fractions were determined by ELISA as described previously [40].

### 3.4. SDS-PAGE and Western Blotting

PSGL-1/mIgG2b purified from cell culture supernatants on goat anti-mouse IgG agarose beads and by chromatographic procedures in large scale were dissolved in 2 × LDS sample buffer (Invitrogen) and incubated at 70 °C for 10 min. For reducing conditions, protein samples were treated with NuPAGE, Sample Reducing Agent 10X (Invitrogen). SDS-PAGE was done under non-reducing conditions using 3%–8% Tris-acetate gradient gels and Tris-acetate SDS running buffer (Invitrogen). Precision protein standard (Hi-Mark, Invitrogen) was applied as reference for protein molecular weight determination. Separated proteins were electrophoretically blotted using iBlot (Invitrogen) in combination with nitrocellulose membranes (Invitrogen). For antibody staining the membranes were blocked with 3% BSA in PBS with 0.2% Tween 20 (PBS-T) and for lectin staining the membranes were blocked with Carbo-Free Blocking solution (SP-5040 Vector laboratories) for 1 h. These membranes were then incubated at room temperature for 1 h with either peroxidase-conjugated anti-mouse IgG (Fc) (diluted 1:20000, Sigma-Aldrich), or mouse anti-human CD162, which recognizes the *N*-terminal of PSGL-1 (diluted 1:1000, BD PharMingen, San Diego, CA, USA), or biotinylated *Maackia amurensis* lectins (MAL-I and MAL-II, 1 μg/mL, Vector laboratories Burlingame, CA, USA), or *Sambucus nigra* bark lectin (SNA, 1 μg/mL, Vector laboratories). Secondary antibodies and reagents for detecting PSGL-1 and lectins were peroxidase-conjugated goat anti-mouse IgG (Fab specific, diluted 1:20000, Sigma-Aldrich) and Avidin (1 μg/mL, Vector Laboratories), respectively. After each incubation, the membranes were washed five times with PBS-T. Bound antibodies and lectins were visualized by chemiluminescence using the ECL kit according to the manufacturer's instructions (GE Healthcare).

### 3.5. LC-MS of O-Glycans Released from Recombinant Proteins

For *O*-glycan analysis, PSGL-1/mIgG2b purified from CP-ext C1, CP-C2, CP-C3-T1, and CP-C4-T1 by protein A affinity chromatography and gel filtration were subjected to reductive β-elimination in solution [41]. However, the PSGL-1/mIgG2b purified from CP-C3(B), CP-C4(B), and all the ST6 stable CHO clones by goat anti-mouse IgG agarose beads were subjected to SDS-PAGE. The proteins were then blotted onto PVDF membranes (Immobilon P membranes, 0.45 μm, Millipore) using a semi-dry method as described previously [41]. Protein bands were visualized using direct blue 71 (Sigma-Aldrich) staining. Bands containing the recombinant PSGL1/mIgG_2b_ were excised and subjected to reductive β-elimination [41].

The *O*-glycans were released from the purified proteins in a solution of 1.0 M NaBH_4_ and 100 mM NaOH for 16 h at 50 °C. In the case of the fusion proteins blotted onto the membrane, the excised strips were incubated with 40 μL of 0.5 M NaBH_4_ and 50 mM NaOH for 16 h at 50 °C. Reactions were quenched with 1 μL of glacial acetic acid, and samples were desalted and dried as previously described [41].

Released *O*-glycans were analyzed by LC-MS using a 10 cm × 150 μm I.D. column, prepared in-house, containing 5 μm porous graphitized carbon (PGC) particles (Thermo Scientific, Waltham, MA, USA). Glycans were eluted using a linear gradient from 0–40% acetonitrile in 10 mM ammonium bicarbonate over 40 min at a flow rate of 10 μL/min. The eluted *O*-glycans were detected using a LTQ ion trap mass spectrometer (Thermo Scientific) in negative-ion mode with an electrospray voltage of 3.5 kV, capillary voltage of −33.0 V, and capillary temperature of 300 °C. Air was used as a sheath gas and mass ranges were defined dependent on the specific structure to be analyzed. The data were processed using the Xcalibur software (version 2.0.7, Thermo Scientific). Glycans were identified from their MS/MS spectra by manual annotation. The annotated structures are submitted to the Unicarb-DB database and will be included in the next release.

## 4. Conclusions

- A number of stable CHO cell lines secreting a mucin-type fusion protein, PSGL-1/mIgG2b, carrying *O*-glycans with different *O*-glycan core saccharides (core 2, 98.0%; core 3, 86.2%; core 4, 31.2%; and ext core 1, 23.5%) have been generated.
- Stable expression of human B3GALT5 in the CP-C3-T1 and CP-C4-T1 clones extended the *O*-glycans with a type 1 outer chain.
- Endogenous ST3GAL(s) activity in CHO cells supports terminal α2,3 sialylation on all *O*-glycan core structures and on both type 1 and type 2 outer chains, while ST6GALT1 could only α2,6-sialylate the type 2 and not the type1 chain.
- The panel of recombinant mucin-like proteins described here, carrying a repertoire of sialylated *O*-glycans based on different core saccharides, will be an important tool for determining the fine *O*-glycan binding specificity of sialic acid-specific microbial adhesins and lectins.
